# Plasma antibodies that cross react to subtype-B and C third variable (V3) region develop in Indian HIV-1 infected iIndividuals with time

**DOI:** 10.1186/1742-4690-9-S2-P55

**Published:** 2012-09-13

**Authors:** R Andrabi, A Gupta, M Bala, K Luthra

**Affiliations:** 1 All India Institute of Medical Sciences (AIIMS), New Delhi, India; 2 Regional STD Teaching Training and Research Centre,Safdarjung Hospital, New Delhi, India

## Background

Subtype-C alone accounts for approximately 50% of global and more than 95% human immunodeficiency virus-1(HIV-1) infection in India. Identification of antigenic epitopes that induce antibodies with cross-clade activity will be crucial to address the HIV-1viral diversity.

## Methods

80 HIV-1 infected drug naive patients were recruited for this study. The study was approved by the institute ethics committee and informed consent was obtained from all the participants. The relative binding of anti-V3 polyclonal plasma antibodies to 35 mer consensus¬-B and C V3 peptides was done by ELISA binding assay. Statistical analysis was performed by Graphpad Prism 5.

## Results

Assessment of the relative binding revealed that 86% (69/80) of the plasma were able to reach an IC50 binding titer with consensus-B V3 peptide with substantially low antibody titers compared to binding with consensus-C V3 (mean IC50 V3-C=12611 versus V3-B=2736) (p<0.0001), implying that although majority of the antibodies were subtype specific, a good proportion of cross reactive anti-V3 antibodies also exist in these plasma (range=1-97%, mean=23%). We observed a strong correlation between percent cross reactive anti-V3 antibodies and days from first diagnosis (n=80: r=0.29 p=0.008) while no such association was found with other clinical and immunological parameters like plasma viral load (n=53: r=0.16 p=0.24), CD4 count (n=80: r=0.10 p=0.34), total plasma IgG levels (n=65: r=-0.09 p=0.45) and eventually with the V3 sequence of donor viruses.

## Conclusion

This is the first study to demonstrate the presence of cross-clade reactive anti-V3 antibodies and their association with time in the plasma of HIV-1 infected Asian Indians from north India.

